# Simultaneous diagnosis of liver PEComa in a family with known Li–Fraumeni syndrome: a case report

**DOI:** 10.1186/s13569-020-00143-7

**Published:** 2020-11-24

**Authors:** María del Mar Galera López, Iván Márquez Rodas, Carolina Agra Pujol, Ángela García Pérez, Enrique Velasco Sánchez, Rosa Álvarez Álvarez

**Affiliations:** 1 Department of Medical Oncology, Hospital General Universitario Gregorio Marañón, Calle Dr. Esquerdo, 46, 28007 Madrid, Spain; 2 CIBERONC, Madrid, Spain; 3 Department of Pathology, Hospital General Universitario Gregorio Marañón, Madrid, Spain; 4 Department of Radiology, Hospital General Universitario Gregorio Marañón, Madrid, Spain; 5 Department of General Surgery, Hospital General Universitario Gregorio Marañón, Madrid, Spain

**Keywords:** Li–Fraumeni syndrome, p53, Sarcoma, Perivascular epithelioid cell tumor, Hepatic lesion

## Abstract

**Background:**

Li–Fraumeni syndrome (LFS) is an autosomal dominant hereditary disease. It is associated with the loss of function of the p53 protein and an increased risk of malignant tumor development at early age. The most frequently detected tumors include breast cancer, sarcomas, leukemia, brain tumors, and adrenocortical carcinomas. While sarcomas account for only 1% of solid tumors, they are more frequently detected in these families.

**Case presentation:**

We report a simultaneous diagnosis of hepatic perivascular epithelioid cell tumor (PEComa), a very rare subtype of sarcoma, in two siblings with a LFS.

**Conclusions:**

The simultaneous diagnosis of PEComa in two siblings presented in this case allowed us to review the frequency of PEComa in this genetic syndrome previously reported, which was very little. Despite its rarity, PEComa must be considered in the differential diagnosis of new-onset liver lesions in patients who were previously diagnosed with LFS.

## Background

Li–Fraumeni syndrome (LFS) is an autosomal dominant hereditary disease, which was described for the first time in 1969 [1]. LFS is associated with abnormalities in the tumor protein p53 gene (*TP53*), a tumor suppressor gene. TP53 protein can delay cell cycle progression, allowing the repair of damaged DNA or initiation of cell apoptosis. Therefore, loss of function contributes to malignant transformation, permitting cells with damaged DNA to survive and proliferate.

The patients with LFS are at an increased risk of developing malignant tumors at early age. Sarcomas, breast cancer, leukemia, brain tumors, and adrenocortical carcinomas are among the most frequently detected types of malignant tumors [2]. Although sarcomas account for less than 1% of all adult cancers, there is an increased risk of developing this type of cancer in the LFS patients, where it represent up to 17% of all diagnosed tumors.

Among the 80 histologic subtypes of sarcomas [3], rhabdomyosarcoma and osteosarcoma have an increased tendency of developing at an earlier age. Other subtypes, such as liposarcoma and leiomyosarcoma, are found in elderly patients [4]. PEComas are a less frequent subtype and the patients with LFS may be at increased risk in developing these tumors.

PEComas belong to a family of tumors that include angiomyolipoma of the kidney (AML), clear cell sugar tumor of the lung (CCST), lymphangioleiomyomatosis of the lung (LAM), myomelanocytic clear cell tumors of the round ligament/sickle cell ligament (CCMMT), and perivascular epithelioid cell tumor not otherwise specified (PEComa-NOS). PEComa-NOS tumors can develop at any anatomical site, including the liver [5].

There are few reported cases of PEComa in LFS patients [6]; however, their relationship with tuberous sclerosis syndrome (TSC) is better known [7].

We report the case of two siblings in the second decade of life, with a previously known LFS, diagnosed with a liver PEComa a few months apart.

## Case presentation

The index case from the family presented in this paper is later described in the article as clinical case no. 2. The patient was diagnosed with rhabdomyosarcoma at the age of one and choroid plexus carcinoma at 3 years of age. Subsequently, LFS was diagnosed with a germline mutation in the *TP53* gene in exon 8 (c.919+1G>A).

Her mother had died of bilateral breast cancer at the age of 42 years. When her older sister was tested (clinical case no. 1), LFS was also diagnosed.

Because of this, both patients were being followed up in the heredofamilial cancer unit of medical oncology.

### Clinical case nº 1

A 29-year-old woman, with no other medical records.

**Fig. 1:**
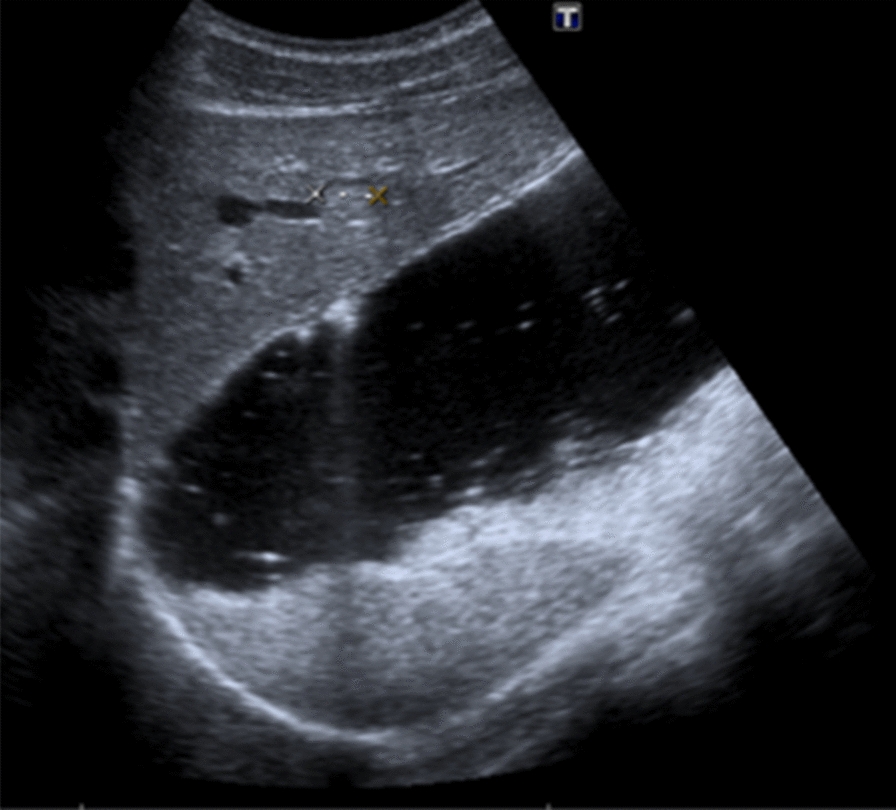
Abdominal ultrasound shows a 1.3 cm lesion in liver segment 2, adjacent to the left suprahepatic branch, isoecogenic with a slightly more hypoecogenic halo

An abdominal ultrasound was performed in February 2019 prior to the start of the fertility treatment and was asymptomatic. The ultrasound identified an isoecogenic solid liver lesion of 1.3 cm in segment 2 with a hypoecogenic halo (Fig. 1).

Due to the known LFS, a study with abdominal magnetic resonance imaging (MRI) was recommended.

**Fig. 2:**
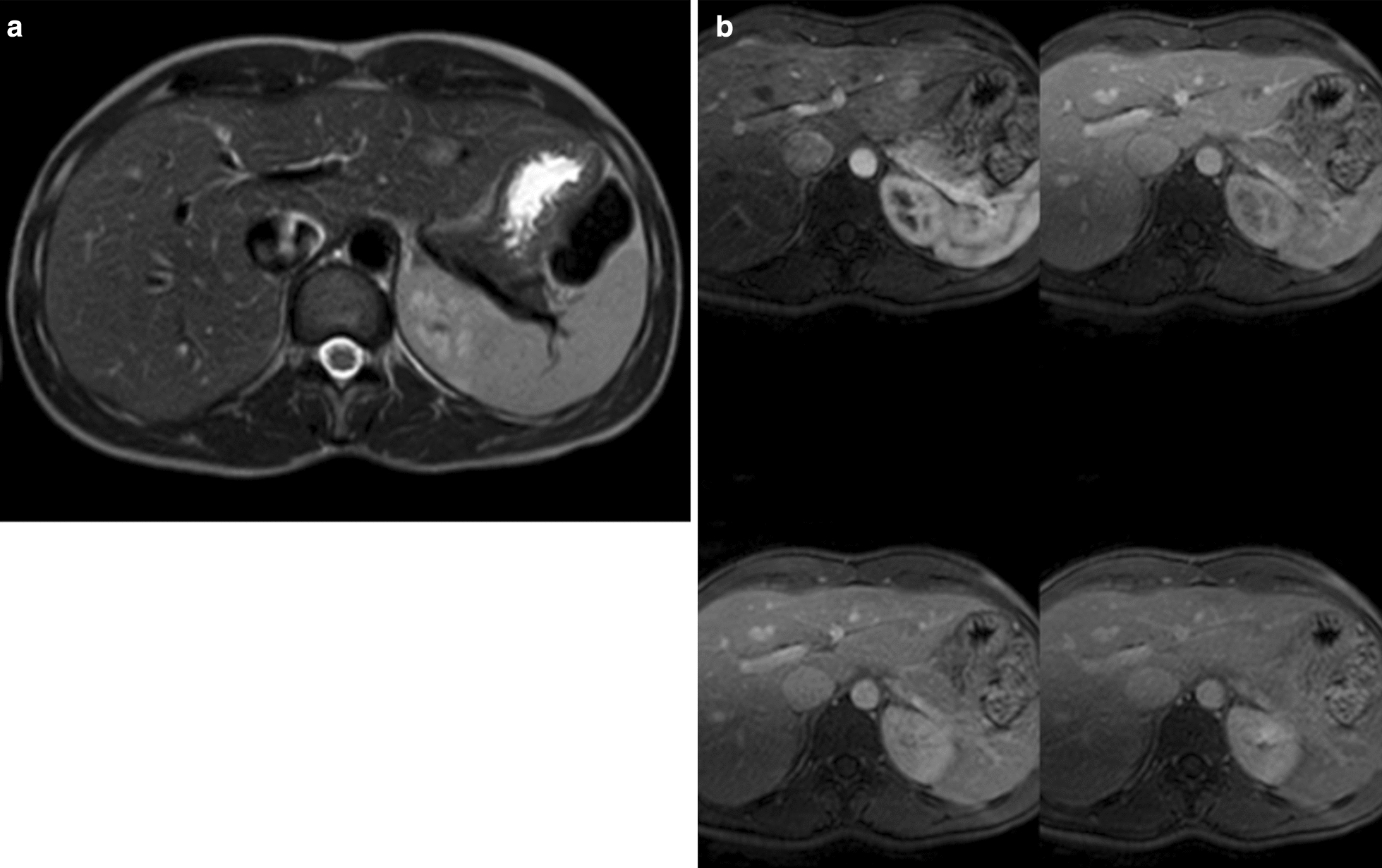
**a**, **b** Abdominal magnetic resonance imaging. In segment 2 adjacent to the left portal branch and below the suprahepatic branch, a focal lesion of 18 mm was identified. The lesion is hypointense in T1, moderately hyperintense in T2 (**a**), although with poorly defined edges and with clear diffusion restriction. In the dynamic study, it is a hypervascular lesion in the arterial phase that washes in the portal and late phases (**b**)

A hypervascular 18 mm lesion was observed in segment 2 of the liver on the MRI scan (Fig. 2). The lesion did not meet the criteria for hemangioma or focal nodular hyperplasia. Hepatic adenoma was considered as the first diagnostic option.

Due to the patient's history, surgical resection was decided by a multidisciplinary committee. The intention was both diagnostic and therapeutic. Finally, on March 2019, resection of segment 2 of the liver was performed.

The histopathological study identified a heterogeneous lesion of 15 mm with free surgical margins. It was a neoplastic proliferation of the mesenchymal lineage, partially encapsulated, composed of medium-sized, polygonal cells with well-defined cell boundaries. The cells with pleomorphic nuclei alternated with clear and eosinophilic granular cytoplasm.

Areas of extracapsular infiltrative growth into the liver parenchyma and necrosis were identified. However, there were no images of vascular invasion. No mitotic figures were identified (0 mitosis/50HPF), and there was a rate of cell proliferation with Ki67 of less than 5%. The immunohistochemical profile was positive for HMB45, Melan-A, and H-Caldesmon.

**Fig. 3:**
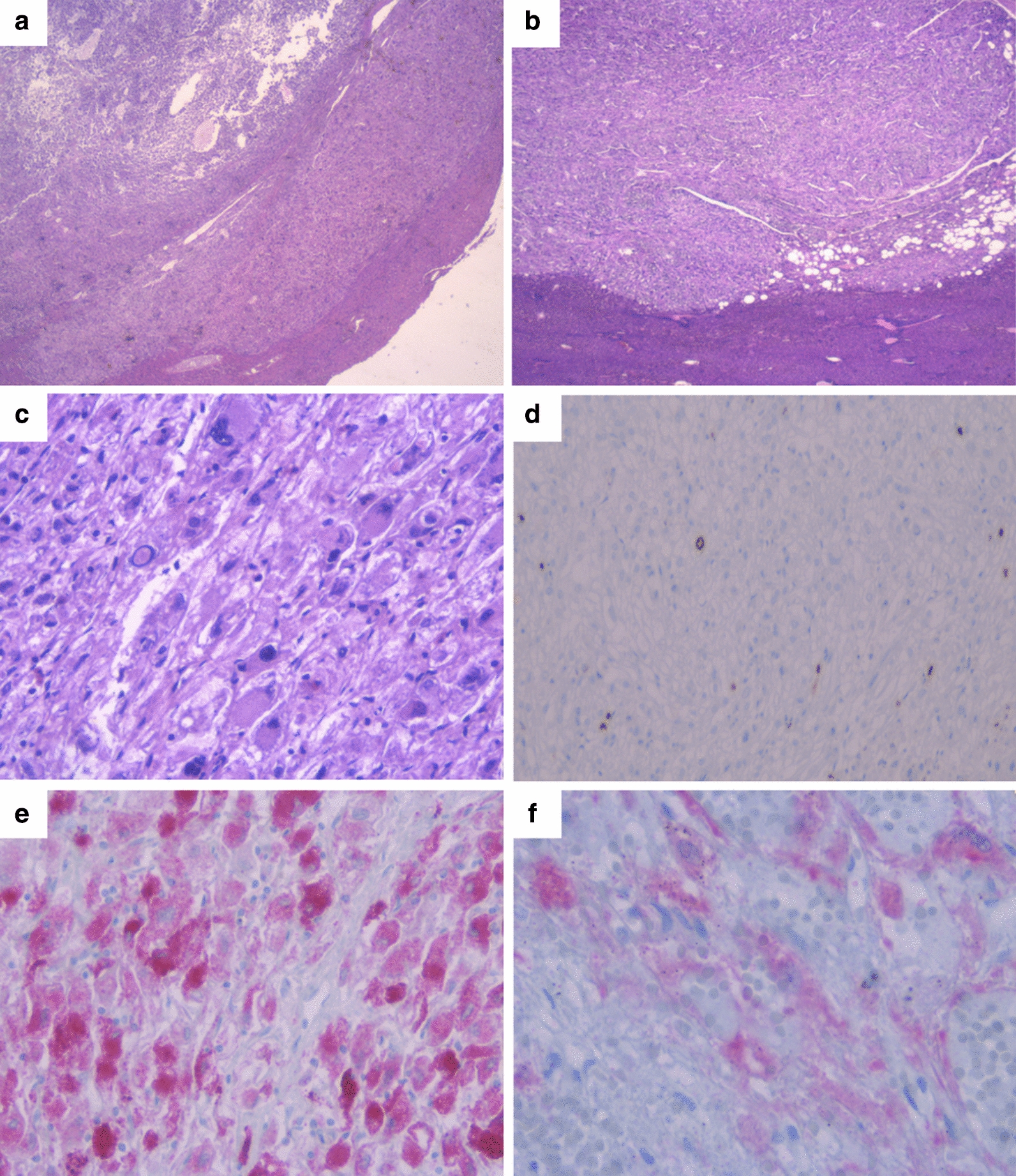
**a**, **b** Pathological anatomy samples of hematoxylin–eosin staining (H&E 1.6×). A heterogeneous neoplastic lesion of 15 mm of mesenchymal lineage, partially encapsulated and circumscribed by hepatic parenchyma. **c** Cells have medium size, are polygonal, with well-defined cell borders, alternating clear cytoplasm and granular eosinophilic (PAS + granules). The cells have markedly pleomorphic nuclei with irregular contours and vesicular chromatin and visible nucleoli (H&E 20×). **d** Cell proliferation rate determined using KI67 is less than 5% by immunohistochemistry (IHC 4×). **e**, **f** IHC staining is positive for HMB45 (**e**) and focally positive for Melan-A (**f**) (IHC 20×)

However, it was negative for S100, SOX10, hepatocyte antigen, CKAE1-AE3, CD34, synaptophysin, TFE3, actin ML, actin HHF, desmin, calponin, and myogenin (Fig. 3).

In addition, it presented an abnormally negative p53 pattern. Morphology and immunohistochemistry were congruent with PEComa.

The tumor presented two risk criteria according to the Folpe et al. classification (infiltrative pattern and necrosis) [8]. Metastasis was not detected on postoperative computed tomography (CT). Therefore, the patient was diagnosed with malignant liver PEComa T1 (1.5 cm) N0 M0, and it was resected with free margins.

Adjuvant treatment with chemotherapy was dismissed in the absence of evidence of benefit in this scenario. The patient remains disease-free at the present time.

### Clinical case nº 2

A 27-year-old male patient.

When the patient was 1 year old, he was diagnosed with an embryonal rhabdomyosarcoma in his left thigh. It was a tumor of 6 × 4 × 3 cm, treated with surgery with a post-surgical stage I, standard risk EpSSG group [9]. After surgery, chemotherapy was initiated following the SIOP Malignant Mesenchymal Tumor 89 protocol [10], containing ifosfamide, vincristine, and actinomycin.

He was diagnosed with a choroidal plexus carcinoma when he was 3 years old. He was operated on two occasions, requiring the insertion of external ventricular drainage for hydrocephalus and peritoneal ventricular bypass. Chemotherapy with the Childrens Cancer Group protocol with an eight-drug chemotherapeutic regimen (vincristine, carmustine, procarbazine, hydroxyurea, cisplatin, cytosine arabinoside, prednisone, and dimethyl-triazenoimidazole-carboxamidea) was administered [11]. Although in complete remission, he presents with intellectual disability and conductive disorders such as sequelae.

**Fig. 4:**
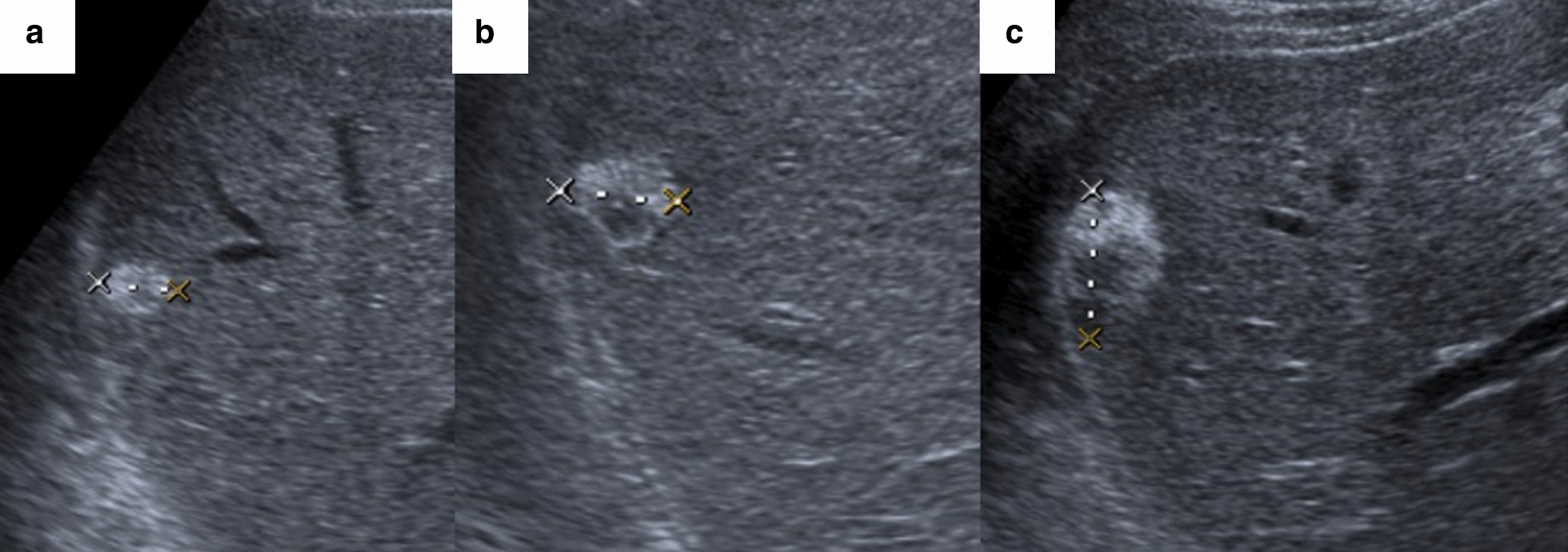
Slowly growing hepatic lesion followed by abdominal ultrasound. A hypercogenic lesion of 13 mm at 2015 (**a**), and of 19 mm at 2017, with an anechoic zone (**b**). At 2019 the lesion was heterogeneous, mixed, and its size was 27 mm (**c**)

**Fig. 5:**
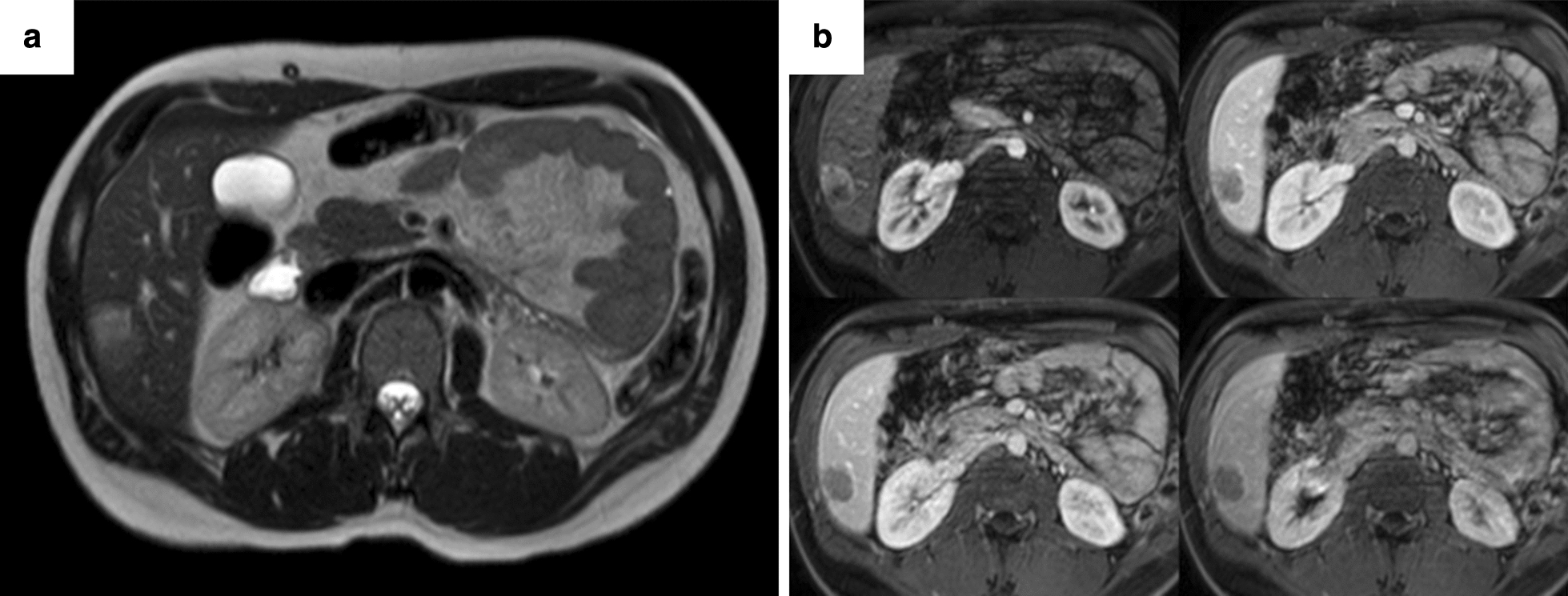
**a**, **b** MRI scans with intravenous contrast. Focal liver injury at segment 6 of 3 × 2.2 cm, hypointense at T1, and discrete hyperintense at T2 with diffusion restriction (**a**). In the dynamic study, it presented enhancement in the arterial phase with washing in the rest of the phases, with a more hypovascular area afterwards (**b**)

A slowly growing focal lesion in hepatic segment 6 was observed on abdominal ultrasound during follow-up (Fig. 4). In June 2019 an MRI was performed to characterize the lesion which had 3 × 2.2 cm of size. It was hypointense in T1 and discreetly hyperintense in T2, with diffusion restriction. In the dynamic study, it presented an enhancement in the arterial phase with washing in the rest of the phases, with a more hypovascular area at the back. The lesion was indeterminate by image, but its hypervascularity could be oriented to a hepatocellular origin (Fig. 5). Metastasis was not detected on a preoperative CT scan.

Due to the personal and family history of the patient, resection of the hepatic segment VI was performed on July 2019. The histological study identified a white nodular lesion measuring 1.9 × 1.2 cm with free surgical margins.

**Fig. 6:**
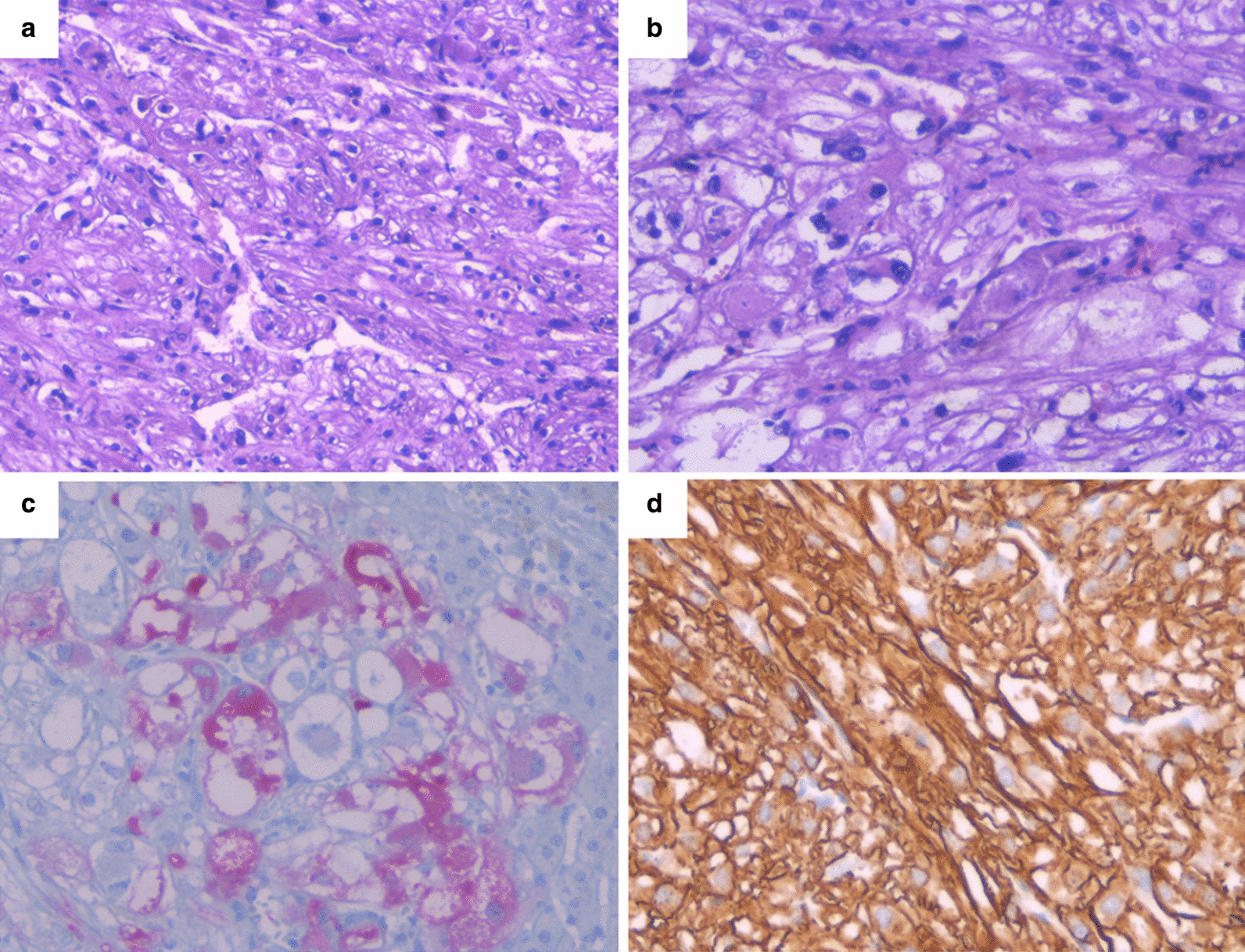
Pathological anatomy samples. **a** Neoplastic proliferation is well-defined but not encapsulated (H&E 10×). **b** Epithelioid-like elements with clear broad cytoplasm and showing foci of mild cytological atypia (H&E 20×). **c** Immunohistochemical staining with positivity for Melan-A and **d** Actin (IHC 20×)

The lesion was formed by a well-defined, although not encapsulated, neoplastic proliferation. Proliferative activity was low (2 mitosis/10 HPF), with a Ki67 rate of 3%. The lesion showed small peripheral extensions towards the surrounding liver, without the presence of distant satellite nodules or vascular or duct invasion phenomena. Immunohistochemical analysis revealed a positivity for Melan-A and Actin, while it was negative for liver and renal markers (CD10 and PAX8) (Fig. 6).

Malignant liver PEComa T1 (1.9 cm) N0 M0 was diagnosed, presenting two risk criteria according to the Folpe et al. classification (infiltrative pattern and mitotic rate > 1/5 HPF).

Similarly to his sister, the patient was not administered adjuvant chemotherapy, and follow-up was continued at our medical oncology department.

## Discussion and conclusions

The subtype of perivascular epithelioid cell tumor or PEComa, is defined as a group of mesenchymal tumors composed of perivascular epithelioid cells, first described by Bonetti et al. in 1992 [12].

These tumors are diagnosed in middle-aged patients (mean age, 38 years). They show a female predominance with a 7:1 ratio, and even excluding the tumors at specific locations by gender, it remains 4:1.

Histologically, PEComas usually present a "nest" architecture, with the formation of trabecule composed of distinctive cells called perivascular epithelial cells or PECs. They are epithelial cells with abundant, granular, clear cytoplasm and small round nuclei.

Immunohistochemically, PEComas are characterized by myomelanocytic differentiation. Therefore, they express melanocytic markers such as HMB-45, Melan-A, or MiTF, simultaneously with muscle markers such as actin, myosin, and calponin [13].

Although the majority of PEComas are benign, a subgroup will present a tendency for recurrence and development of metastasis. Indeed, in the WHO 2013 classification, they are categorized within the subgroup of malignant tumors with uncertain differentiation [5]. Using the risk criteria identified by Folpe et al. (Table 1), an adapted risk stratification was proposed that classified the PEComas either as benign, of uncertain malignant potential, or malignant (Table 2) [8].

**Table 1:** Risk factors Folpe et al. [13]

High risk features Folpe et al.
Size > 5 cm
Infiltrative growth pattern
High nuclear grade and cellularity
Mitotic rate > 1/5 HPF
Necrosis
Vascular invasion

**Table 2:** PEComa prognostic classification adapted from Folpe et al. [8]

Prognosis	Risk factors
Benign	< 2 risk factors Size < 5 cm
Uncertain malignant potential	Size > 5 cm with no other risk criteria or only cell pleomorphism/multi-nuclear giant cells
Malignant	2 or more risk factors

PEComas present genetic heterogeneity. Sporadic mutations in tuberous sclerosis complex 1 (TSC1) and 2 (TSC2) are detected at high frequency in PEComas, with the secondary activation of the mTOR pathway. TFE3 rearrangements have also been detected in PEComas that did not present TSC mutations, suggesting an alternative tumorigenesis pathway for which the exploration of other therapeutic alternatives to mTOR inhibitors was suggested. The mutations in *TSC2* and *TP53* genes coexist and suggest a more aggressive behavior [14].

The role of *TP53* in PEComa oncogenesis requires further research. In a TSC patient with a renal AML with epithelioid malignant transformation, a missense *TP53* mutation at codon 249 was detected. Because *TP53* mutation was detected only in the malignant components, a possible role of *TP53* in the malignant transformation of AML was proposed [15]. In another TSC-associated epithelioid AML, focal areas of p53 immunoreactivity and a single nucleotide polymorphism in the exon 6 were detected [16]. Pan et al. studied the molecular genetics in nine PEComas cases. In addition, they detected chromosomal loss of 17p in six cases (the tumor suppressor gene around this region is TP53) and immunohistochemical staining for TP53 was positive only in the TSC case with an epithelioid AML [17]. Epithelioid angiomyolipoma, also called pure epithelioid PEComa (PEP), is associated with aggressive behavior. In a series of eight PEPs (five from kidneys, one from heart, liver, and the uterus, each), the p53 nuclear staining was stronger than that in classic AMLs. The p53 mutation analyses by direct sequencing of exons 5 to 9 showed 4 mutations in 3 of 8 PEPs, which included 2 missense mutations in exon 5 (one involving codon 165 and the other involving codon 182) in a hepatic PEComa and 2 silent mutations in 2 renal PEPs [18].

Although most PEComas are sporadic, a small subgroup may be associated with genetic syndromes. These tumors are well known to be associated with TSC, an autosomal dominant multisystemic neurocutaneous disorder. TSC is characterized by the development of benign tumors in multiple organs. It affects the central nervous system in almost all cases, with epilepsy in 80–90% of patients and loss of intellectual capacity in more than 50% of patients.

Tuberous sclerosis presents germline mutations with the loss of function in the tumor suppressor genes *TSC1* and *TSC2*, which are the negative regulators of the mTOR signaling pathway. The loss or inactivation of either of these two genes results in increased expression of RheGTP, which interacts directly with mTORC1, causing its activation. This syndrome is associated with an increased risk of malignancy, both intracranial tumors (subependymal nodes and subependymal giant cell astrocytomas) and a variety of extracranial tumors, including renal angiomyolipoma and lymphangioleimiomatosis. Most PEComas non-RMA or LAM tumors are sporadic, with only a small subset associated with TSC [19].

It is less well known whether the TSC is associated with LFS. Although its relationship with increased frequency in sarcoma is known, few cases of PEComa have been reported [6].

The carriers of LFS, have an increased risk of developing malignant tumors. LFS individuals have one inherited abnormal copy of the *TP53* gene and the second allele of *TP53* is either mutated or deleted somatically at the tumor site, leaving cells with no functional gene product. *TP53* is a tumor suppressor gene, and the gene product TP53 can delay cell cycle progression for DNA repair or apoptosis. In the absence of the functional TP53, cells containing damaged DNA can survive and proliferate, which contributes to malignant transformation.

The elevated incidence of soft tissue sarcomas in LFS patients indicates that a germline mutation in *TP53* and a deficiency in p53 function allow for muscle cell transformation [20]. Somatic *TP53* mutations have been proposed to be relatively early events in other forms of sarcomas [21], further suggesting that muscle cells must overcome an early p53-dependent blockade during tumor initiation.

The most common tumors include osteosarcomas and soft tissue sarcomas, breast cancer, leukemia, brain tumors, and adrenocortical carcinomas. In childhood, the most common cancers in addition to adrenocortical carcinoma, are choroidal plexus carcinoma, gliomas, and medulloblastoma. Other tumors that have an increased risk are melanoma, stomach, colon, pancreas, esophagus, and gonadal germ cell tumors.

For individuals belonging to families that meet the classical or Chompret criteria, the *TP53* mutation test is recommended. It will also be considered for individuals with breast cancer under 31 years of age with negative results for breast cancer gene *BRCA* 1 and 2, diagnosis of adrenocortical carcinoma, sarcoma in childhood other than Ewing’s sarcoma or choroidal plexus carcinoma, regardless of family history.

The lifelong risk of cancer development in these patients reaches 49% in women and 21% in men under the age of 30. Throughout their lives, the risk increases to almost to 100% in women and 73% in men. Moreover, 15%, 4%, and 2% of individuals develop 2, 3, and 4 tumors, respectively.

Regarding the best follow-up strategy for patients with known LFS, several centers have also studied the role of whole-body magnetic resonance imaging for early detection of soft tissue sarcoma and osteosarcoma [22]. It is currently recommended for the management of p53 mutation carriers with 2B evidence by NCCN guidelines [23]. More generally accepted measures include annual skin and neurological examinations, annual MRI breast scans, and endoscopies every 2–5 years from age 25 (or 5 years prior to the first family diagnosis).

In the meta-analysis of 13 observational cohorts with 578 participants, 42 cancers were diagnosed, 35 of which were located and treated with curative intent. However, false positives were detected in 173 patients (29.9%), which required additional imaging tests and biopsies [24].

Regarding to the treatment of PEComas, surgical resection is the only curative treatment.

In patients with unresectable or metastatic tumors, responses to mTORC1 inhibitors have been reported. Responses were initially described in angiomyolipomas and lymphangioleiomyomatosis associated with tuberous sclerosis [25, 26] and later in non-tuberous sclerosis-associated PEComas, both in isolated cases and case series [27–31]. In a retrospective review by Sanfilippo et al. of 53 patients diagnosed with PEComa, mTOR inhibitors were the most active agents with an overall response rate of 41% and a median progression-free survival of 9 months. Notably, a subset of responding patients experienced a response longer than 1 year (28.2%) [32]. The phase II trial AMPECT recently reported results with nab-sirolimus in 34 patients with PEComa. The overall response rate was 39%, and the median duration of responses was not reached (more than 25.8 months). This response was more durable than that observed with other mTOR inhibitors. The treatment was also safe and well tolerated. Patients with TSC2 mutations were more likely to have a nab-sirolimus response (89% of the patients) [33].

The use of standard chemotherapy agents has been shown to have little evidence of the efficacy in this disease, and their role is limited. For the standard agents used for the treatment of soft tissue sarcomas such as anthracyclines and ifosfamide, only the anecdotal responses or disease stabilization have been reported in PEComas [34].

In the retrospective review reported by the Istituto Nazionale Tumori and the Italian Rare Cancer Network, rates of discrete responses to other chemotherapy agents such as combinations with gemcitabine were described in only 33% of patients (2 stabilizations and 2 partial responses of 15 patients) and of short duration (progression-free survival of 3.4 months) [32].

Treatment with antiangiogenic agents may have a role in the treatment of this disease, taking into account those PEComas with TFE3 translocations. In the same Italian series, partial response rates of 8% and stabilization of 75% of 12 patients are described [35]. A clinical case of a patient responding to a combination of mTOR inhibitors and antiangiogenics (sirolimus and sorafenib) has also been described as an option to be considered pending completion of study [35].

Because of this lack of treatment effectiveness in already metastatic patients, early diagnosis is important.

In this reported case, abdominal imaging tests allowed for early detection of the disease and its treatment with curative intent. Despite the low frequency of PEComas with hepatic localization [36], we should consider them as one of the possible tumors in the differential diagnosis of new-onset liver lesions. The long-term impact of these early diagnoses is not yet known, and neither is the adequate frequency of MRI follow-up in p53 mutation carriers. Long-term studies that address these issues are needed.

Despite the rarity of PEComas, these tumors can arise in the context of an increased risk of sarcoma development at LFS. For this reason, we report this case of two close age siblings, with the same diagnosis in a short period of time. The scarcity of reported cases of LFS and PEComa does not allow us to find an association with the mutation presented by this family.

Although we still cannot be certain if the increased number of imaging tests, such as full MRI or abdominal ultrasound have an impact on survival, there are already some clinical guidelines that consider it. In the two patients presented in this case, early detection has made surgical resection possible, the only curative option for this entity, which is not very sensitive to systemic treatments. We expect long-term studies to analyze the benefit of follow-up through these imaging tests.

## Data Availability

The datasets during and/or analyzed during the current study are available from the corresponding author upon reasonable request.

## Abbreviations

AML: Angiomyolipoma of the kidney
CCST: Clear cell sugar tumor of the lung
CT: Computed tomography
H&E: Hematoxylin–eosin
IHC: Immunohistochemistry
HPF: High-power field
LAM: Lymphangioleiomyomatosis of the lung
LFS: Li–Fraumeni syndrome
MRI: Magnetic resonance imaging
PEComa: Perivascular epithelioid cell tumor
PEComa-NOS: Perivascular epithelioid cell tumor not otherwise specified
PEP: Pure epithelioid PEComa
TP53: Tumor protein p53 gene
TSC: Tuberous sclerosis
TSC1: Tuberous sclerosis complex 1
TSC2: Tuberous sclerosis complex 2
